# The Design and Construction of K11: A Novel **α**-Helical Antimicrobial Peptide

**DOI:** 10.1155/2012/764834

**Published:** 2012-02-16

**Authors:** Huang Jin-Jiang, Lu Jin-Chun, Lu Min, Huang Qing-Shan, Li Guo-Dong

**Affiliations:** ^1^State Key Laboratory of Genetic Engineering, Institute of Genetics, College of Life Science, Fudan University, Shanghai 200433, China; ^2^Shanghai Hi-Tech United Bio-Technological Research & Development Co., Ltd, Shanghai 201206, China

## Abstract

Amphipathic **α**-helical antimicrobial peptides comprise a class of broad-spectrum agents that are used against pathogens. We designed a series of antimicrobial peptides, CP-P (KWKSFIKKLTSKFLHLAKKF) and its derivatives, and determined their minimum inhibitory concentrations (MICs) against *Pseudomonas aeruginosa*, their minimum hemolytic concentrations (MHCs) for human erythrocytes, and the Therapeutic Index (MHC/MIC ratio). We selected the derivative peptide K11, which had the highest therapeutic index (320) among the tested peptides, to determine the MICs against Gram-positive and Gram-negative bacteria and 22 clinical isolates including *Acinetobacter baumannii*, methicillin-resistant *Staphylococcus aureus, Pseudomonas aeruginosa, Staphylococcus epidermidis,* and *Klebsiella pneumonia*. K11 exhibited low MICs (less than 10 *μ*g/mL) and broad-spectrum antimicrobial activity, especially against clinically isolated drug-resistant pathogens. Therefore, these results indicate that K11 is a promising candidate antimicrobial peptide for further studies.

## 1. Introduction

 The extensive and intensive use of antibiotics has led to the emergence of resistant strains of bacteria. During the last few years, only three new types of antibiotics have been developed for clinical use [1]. Antimicrobial peptides have been identified from a wide variety of sources, including bacteria, insects, plants, and animals [2, 3]. Most native antimicrobial peptides are effective against a broad spectrum of pathogens but can also be toxic to normal cells [4].

The intensive study of the structure and function of antimicrobial peptides has led to the development and clinical application of many peptides with enhanced activity and low toxicity; these peptides have been developed through sequence splicing, amino acid substitution, and changing the ratio of hydrophobic amino acids [5–7]. Among them, *α*-helical antimicrobial peptides are one of the most studied types [8]. CP26, composed of the N-terminal 8 amino acid residues of cecropin A1 and the N-terminal 18 amino acid residues of melittin, has high antimicrobial activity and low toxicity [9]. P18, composed of 8 amino acid residues of the N-terminus of cecropin A1 and the N-terminal 12 amino acid residues of magainin 2, showed satisfactory antimicrobial activity and no toxicity [10].

In this study, CP26 and P18 were chosen as template peptides for the synthesis of CP-P, which is composed of amino acids 1 to 11 at the N-terminus (KWKSFIKKLTS) of CP26 and amino acids 10 to 18 at the C-terminus (KFLHLAKKF) of P18. CP-P was modified to yield a number of peptides with single amino acid substitutions at different sites on the polar or nonpolar faces, and their therapeutic indices (MIC/MHC) were evaluated against *Pseudomonas aeruginosa*. The in vitro activities of the selected peptides with high therapeutic indices were evaluated against a set of multiresistant clinical isolates of both Gram-positive and Gram-negative pathogens. The results indicate that the modifications changed the antimicrobial activities and hemolytic toxicities. Among the derivatives, K11 exhibited a high therapeutic index and may have potential for use in further studies.

## 2. Materials and Methods

### 2.1. Bacterial Strains

 Twenty-two clinical isolates and night standard laboratory strains were used in this study: 4 *Acinetobacter baumannii*, 4 *Pseudomonas aeruginosa, *3 *Staphylococcus epidermidis*, and 6 *Klebsiella pneumonia* (Changhai Hospital, Shanghai, China); 5 methicillin-resistance* Staphylococcus aureus* (Ruijin Hospital, Shanghai, China); *Staphylococcus aureus *CMCC26003; *Bacillus subtilis* DB430;*Bacillus pumilus *CMCC63202; *Micrococcus luteus* CMCC28001; *E. coli *ATCC8099; *Klebsiella pneumoniae *CMCC46117;*Salmonella paratyphi *B CMCC50094; *Pseudomonas aeruginosa *CMCC10104; *Micrococcus *S1.634. In addition, *Pseudomonas aeruginosa *CMCC10104 was used during the design of the peptides for evaluating antimicrobial activities. All strains were cultured on Mueller-Hinton medium.

### 2.2. Design of Peptides

CP-P was designed by splicing the sequences of CP26 and P18. S16, which has a single amino acid change at the 16th site of CP-P, served as a template peptide from which several peptides were derived by single amino acid substitution at different sites. All of the peptide sequences are listed in Table 1.

### 2.3. Synthesis and Purification of Peptides

The syntheses of the peptides CP26, P18, CP-P, S16, and the S16 derivatives (a total of 23) were carried out by solid-phase peptide synthesis (SPPS) [11]. The crude peptides were purified by RP-HPLC using a Kromasil C18-5 column at a flow rate of 1 mL/min with a linear AB gradient (1% acetonitrile/min); mobile phase A was 0.1% trifluoroacetic in 100% water, and mobile phase B was 0.1% trifluoroacetic in 100% acetonitrile. The identities of the purified peptides were confirmed by mass spectrometry.

### 2.4. Circular Dichroism (CD)

CD spectra were obtained with a Jasco J-710 instrument (Jasco, Tokyo, Japan) utilizing quartz cells with a 2 mm path length and peptide concentrations of 100 *μ*M in a 10 mM sodium phosphate buffer with a pH of 7.5 containing 50% trifluoroethanol. Each spectrum was obtained from an average of 5 pairs of duplicates, and the percentage helicity of each peptide was determined [12].

### 2.5. Measurement of the Antibacterial Activity

The antibacterial activities of the different peptides were evaluated using the minimum inhibitory concentration (MIC). The process followed the standard microtiter dilution method in LB medium without salt. Briefly, bacteria were grown overnight in LB at 37°C and diluted in the same medium. Then, 100 *μ*L of the medium was dispensed into each well of a 96-well plate. The number of bacteria was between 10^4^ and 10^5^ CFU/mL. Serial dilutions of peptides were performed consecutively in each well in 10 *μ*L. Plates were incubated at 37°C for 24 h; then, the OD_620_ was measured to determine the MIC of each peptide [13].

### 2.6. Measurement of Hemolytic Activity (MHC)

The MHCs were measured by the method as described in Chen et al. [7]: peptides were added to 1% human erythrocytes in phosphate-buffered saline (0.08 mol/L NaCl, 0.043 mol/L Na_2_HPO_4_, 0.011 mol/L KH_2_PO_4_) and then kept at 37°C for 1 h in microtiter plates. The supernatants were collected by centrifugation (800 g), and the OD_562_ of each supernatant was determined. We chose a 0.1% Triton X-100 solution as the positive control because this solution causes the total release of hemoglobin. We defined the MHC as the highest peptide concentration that caused no detectable release of hemoglobin (the ratio of the OD_562_ for the peptide solution to the OD_562_ for the positive control should be less than 1%).

## 3. Results

### 3.1. Design and Construction of Peptides

**Figure 1:**
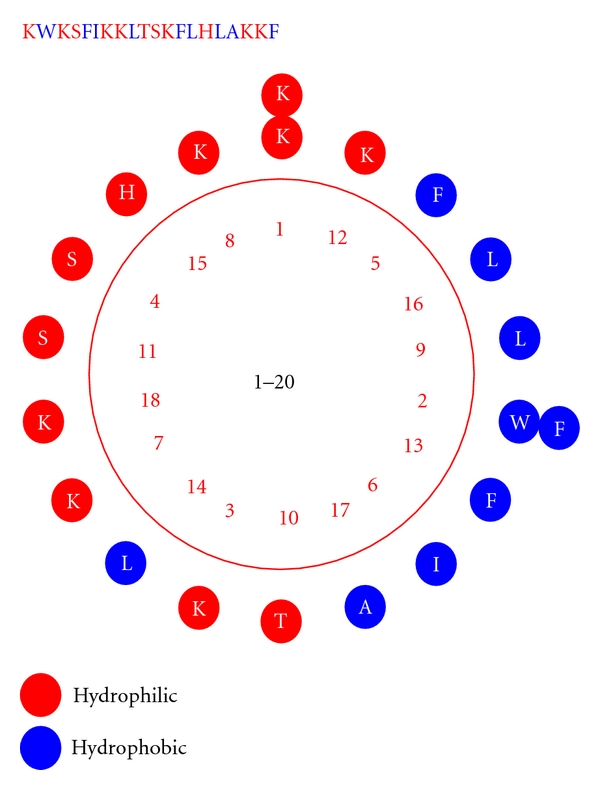
Helical wheel structure and sequence of peptide CP-P.

All of the peptides were designed based on a CP-P template, which had a higher therapeutic index than P18 and P26. S16, a derivative of CP-P with only one amino acid substitution at the 16th site, exhibited an 8-fold on the therapeutic index. This result indicates that a polar amino acid substitution on the nonpolar face could decrease the amphipathicity and influence the antimicrobial activity to some extent.  Figure 1 [14] shows the helical wheel structure of CP-P, which illustrates the polar and non-polar faces of CP-P clearly. We chose specific sites for single amino acid substitutions, constructed a series of peptides and then calculated the therapeutic index of all of the derivatives.

### 3.2. The Purification and Determination of the Molecular Confirmation of the Peptides

**Figure 2:**
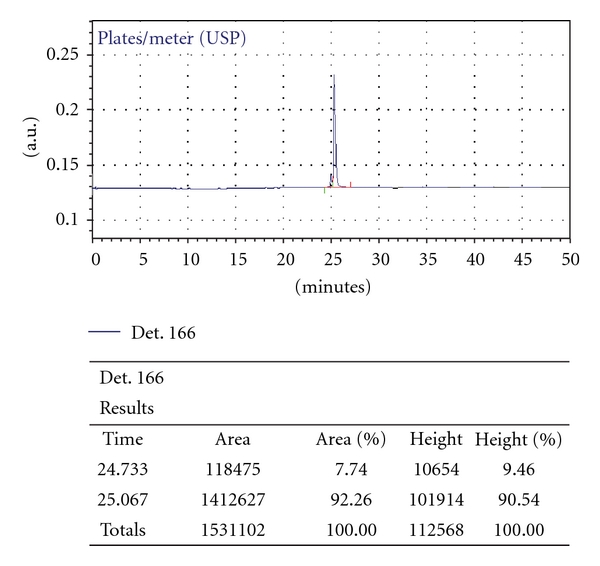
Analysis of the purity of peptide S16 by HPLC

After purification, the designed peptides were analyzed by RP-HPLC, which showed that their purities were greater than 90%. Figure 2 shows that peptide S16 reached a purity of 92.3% after purification. The S16 peak was collected and analyzed by mass spectrometry. Table 1 shows that the mass spectrometry results of the synthetic peptides matched well with those calculated by BioPerl [15].

### 3.3. Evaluation of the Designed Peptides

 After the mutation of the 16th site to serine (S16), the MIC decreased to 1/4 of that of CP-P (Table 2). Table 2 also provides information about other peptides that are based on modifications of S16 by amino acid substitutions. Among these 21 peptides, peptide K11 showed the highest efficacy, with an MIC of 1.6 *μ*g/mL, an MHC of more than 500 *μ*g/mL and a therapeutic index of 320.

 The hydrophobic moments were determined with the HeliQuest web server [16], which can be used to evaluate amphipathicity. The percent helix values were determined based on CD spectra.

### 3.4. Antimicrobial Activity Against Clinical *Acinetobacter Baumannii* Isolates

After comparison, three designed peptides, S16, K11, and P18, were chosen to use in MIC tests on four strains of clinically isolated *Acinetobacter baumannii*.  Table 3 illustrates that peptide K11 had the lowest MICs for the four strains of *Acinetobacter baumannii*; these MICs were between 0.8 and 3.25 *μ*g/mL. Upon consideration of this result, we evaluated the antimicrobial activity of K11 against clinical isolates of both Gram-positive and Gram-negative pathogens.

### 3.5. Antimicrobial Activity of Peptide K11 Against Different Bacteria

Similar to the results above, peptide K11 had a lower MIC than the other designed peptides.  Table 4 shows the MICs of K11 against different bacteria, revealing that K11 exhibited broad-spectrum antimicrobial activity.

### 3.6. Antimicrobial Activities of Peptide K11 Against Clinical Isolates

The results of MIC tests on different clinical bacterial isolates can be found in Table 5. The clinically isolated bacteria included 5 strains of MRSA, 4 strains of *Pseudomonas aeruginosa*, 3 strains of *Staphylococcus epidermidis* and 6 strains of *Klebsiella pneumonia*. Peptide K11 exhibited a low MIC for almost all of the strains of bacteria, which indicated that this peptide has a high antimicrobial activity against several clinically isolated drug-resistant bacteria.

## 4. Discussion

To design novel antimicrobial peptides with enhanced biological properties, we searched native antimicrobial peptides and selected CP26 and P18 as frameworks. CP26 and P18 have been reported to be two *α*-helical peptides with high antimicrobial activities. Starting with CP26 and P18, we designed and constructed a dozen novel peptides by sequence splicing and amino acid substitution. Several of these peptides had lower MICs and higher therapeutic indices than CP26 and P18. Among them, K11 showed the maximum antimicrobial activity and minimum eukaryotic cell toxicity, with an MIC 1/16 of that of P18 and a 32-fold higher therapeutic index than P18.

Recent studies on *α*-helical antimicrobial peptides have revealed that the amphipathic structure plays a primary role in the antimicrobial activity of these compounds. The reduction of antimicrobial activity resulting from the enhanced ability of self-aggregation caused by increasing amphipathicity has been reported [17]. The mechanism responsible for this effect may be the reduction of the effective molecular number after self-aggregation. In our study, we used HeliQuest to calculate the hydrophobic moment of peptides to evaluate the amphipathicity and came to the conclusion that high amphipathicity reduced the therapeutic index. S16 has a lower hydrophobic moment than CP-P (0.596 to 0.648) and was found to have a higher therapeutic index (160 to 20). Peptides L10 and D11 have high hydrophobic moments (0.632 and 0.631, resp.) and exhibited low therapeutic indices (<20). The MICs increased from 3.125 *μ*g/mL for S16 to 12.5 *μ*g/mL and 25 *μ*g/mL, and the MHCs decreased from >500 *μ*g/mL for S16 to <125 *μ*g/mL. These results confirm the conclusion above, but high hydrophobic moments may also lead to increased antibacterial activities and decreased hemolytic activities. The results agree with the results of other studies that there is a threshold hydrophobicity at which optimal antimicrobial activity can be achieved [18]. In this study, K11 reached a therapeutic index of 320 and has a high hydrophobic moment (0.642).

We found that substitutions at certain specific sites (9, 11, and 13 from the N-terminus) can change the antimicrobial activity of S16 more effectively. The 9th residue of S16 is L, which is on the nonpolar face. If a polar amino acid were substituted at this site, there could be an increase in the MIC. R9, S9, and K9 are three derivatives that showed high MICs (>50 *μ*g/mL) and decreased hydrophobic moments (0.494, 0.529, and 0.495, resp.). The substitutions at the 11th and 13th sites with opposite polar amino acids can also lead to significant increases in the MIC and decreases in the amphipathicity. We think that this structural implication should be investigated more closely in future studies.

The net positive charge on the polar face is important for the antimicrobial and hemolytic activities of antimicrobial peptides [19]. We constructed K11 by changing S to K at position 11 on the polar face of S16. This one addition to the net charge resulted in K11 showing the best biological properties among the tested peptides.

In conclusion, our results showed that the therapeutic index of a peptide depends on several factors. Modification of specific sites can change the amphipathicity to different extents, which can be used to predict the antimicrobial activity. In our study, we found a highly effective peptide, S16, and used it as template to establish a series of peptides with single amino acid substitutions. Among these peptides, K11 showed strong therapeutic action against antibiotic-resistant clinical isolates of both Gram-positive and Gram-negative bacteria, making it a promising antimicrobial peptide candidate for further study, especially in vivo studies and clinical tests.

## Figures and Tables

**Table 1:** Peptide sequences.

Peptide	Sequence	MW (Calc)^a^	MW (Deter)^b^
CP26	KWKSFIKKLTSAAKKVVTTAKPLISS-NH_2_	2859.7	2860.5
P18	KWKLFKKIPKFLHLAKKF-NH_2_	2299.4	2300.5
CP-P	KWKSFIKKLTSKFLHLAKKF-NH_2_	2480.1	2479.1
S16	KWKSFIKKLTSKFLHSAKKF-NH_2_	2453	2452.1
F2	KFKSFIKKLTSKFLHSAKKF-NH_2_	2415	2414.1
N3	KWNSFIKKLTSKFLHSAKKF-NH_2_	2439.9	2439.1
K6	KWKSFKKKLTSKFLHSAKKF-NH_2_	2469	2468.1
N7	KWKSFINKLTSKFLHSAKKF-NH_2_	2439.9	2438.9
A9	KWKSFIKKAKTSFLHSAKKF-NH_2_	2411.9	2410.7
K9	KWKSFIKKKTSKFLHSAKKF-NH_2_	2469	2468.1
S9	KWKSFIKKSTSKFLHSAKKF-NH_2_	2427.9	2426.7
R9	KWKSFIKKRTSKFLHSAKKF-NH_2_	2497	2496.1
A10	KWKSFIKKLASKFLHSAKKF-NH_2_	2424	2424.1
L10	KWKSFIKKLLSKFLHSAKKF-NH_2_	2466	2465.1
D11	KWKSFIKKLTDKFLHSAKKF-NH_2_	2482	2481.7
K11	KWKSFIKKLTKKFLHSAKKF-NH_2_	2495.1	2494.4
L11	KWKSFIKKLTLKFLHSKKKF-NH_2_	2480.1	2479.2
A13	KWKSFIKKLTSKALHSAKKF-NH_2_	2377.9	2378.1
K13	KWKSFIKKLTSKKLHSAKKF-NH_2_	2435	2434.3
K17	KWKSFIKKLTSKFLHSKKKF-NH_2_	2511.1	2510.2
D18	KWKSFIKKLTSKFLHSADKF-NH_2_	2440.9	2440.2
N18	KWKSFIKKLTSKFLHSANKF-NH_2_	2439.9	2440
N20	KWKSFIKKLTSKFLHSAKKN-NH_2_	2420.9	2419.6

^a^Calculated using BioPerl.

^b^Determined using mass spectrometry.

**Table 2:** The MICs, MHCs, and therapeutic indices of the designed peptides.

Peptide	MIC (*μ*g/mL)	MHC (*μ*g/mL)	Therapeutic index	Hydrophobicity	Hydrophobic moment	%Helix	Net charge
P18	25	250	10	0.486	0.405	N	7
CP-P	12.5	250	20	0.411	0.648	63.2	7
S16	3.125	>500	160	0.323	0.596	71.8	7
F2	12.5	250	20	0.3	0.574	N	7
N3	12.5	N	N	0.343	0.599	72.5	6
K6	12.5	N	N	0.184	0.471	N	8
N7	12.5	>500	40	0.343	0.586	72.1	6
K9	>50	N	N	0.189	0.495	51.5	8
S9	50	>500	10	0.236	0.529	68.2	7
R9	>50	N	N	0.188	0.494	N	8
A9	25	>500	20	0.254	0.496	64.6	7
L10	12.5	<125	10	0.395	0.632	72.1	7
A10	12.5	250	20	0.326	0.597	72.5	7
D11	25	>500	20	0.287	0.631	71.6	6
K11	1.6	>500	320	0.276	0.642	67.4	8
L11	>50	N	N	0.411	0.513	N	7
A13	6.25	>500	80	0.249	0.522	45.9	7
K13	>50	N	N	0.184	0.457	62.6	8
K17	12.5	>500	40	0.258	0.551	58.7	8
D18	25	>500	20	0.343	0.587	51.4	5
N18	6.25	>500	80	0.343	0.58	37.2	6
N20	6.25	>500	80	0.204	0.483	N	7

Note: “N” signifies that the data were not determined.

**Table 3:** MIC tests on four strains of clinical *Acinetobacter baumannii *isolates.

Peptide	MIC (*μ*g /mL)			
	b-01	b-02	b-03	b-04
P18	6.25	25	12.5	12.5
S16	3.125	6.25	6.25	3.125
K11	0.8	3.125	3.25	1.6

**Table 4:** MICs of peptide K11 for different bacteria.

	Bacteria	MIC (*μ*g/mL)
Gram-positive bacteria	*Staphylococcus aureus * CMCC26003	0.5
	*Bacillus subtilis * DB430	2
	*Bacillus pumilus * CMCC63202	0.5
	*Soluble wall Micrococcus * S1.634	1
	*Micrococcus luteus * CMCC28001	2

Gram-negative bacteria	*E. coli * ATCC8099	0.5
	*Klebsiella pneumoniae * CMCC46117	2
	*Salmonella paratyphi* B CMCC50094	2
	*Pseudomonas aeruginosa * CMCC10104	4

**Table 5:** MICs of K11 for clinically isolated bacteria.

Bacteria	MIC (*μ*g/mL)
MRSA (5)	0.25−4
*Pseudomonas aeruginosa* (4)	1.0−8.0
*Staphylococcus epidermidis* (3)	0.5−8
*Klebsiella pneumonia* (6)	0.5−4.0
